# From classroom to bedside: integration of the basic science curriculum in medical teaching

**DOI:** 10.1590/1516-3180.2013.1314730

**Published:** 2013-08-01

**Authors:** Alessandro Wasum Mariani, Paulo Manuel Pêgo-Fernandes

**Affiliations:** I MD. Thoracic Surgeon, Instituto do Coração (InCor), Hospital das Clínicas (HC), Faculdade de Medicina da Universidade de São Paulo (FMUSP), São Paulo, Brazil.; II MD, PhD. Associate Professor, Discipline of Thoracic Surgery, Instituto do Coração (InCor), Hospital das Clínicas (HC), Faculdade de Medicina da Universidade de São Paulo (FMUSP), São Paulo, Brazil.

Around the world, universities have been rethinking not only the medical curriculum but also the entire way of teaching medicine. The enormous torrent of new knowledge, medical specialization, ever-faster entry of technology and even the concepts of evidence-based medicine have made it essential to discuss again and reorganize the medical curriculum.

In many medical schools, the undergraduate medical curriculum can classically be divided into basic sciences, clinical sciences and clerkship (internship). The increases in the length of time spent on clerkship that have been instituted in many medical schools is a sign of change in teaching and a clear example of a significant curricular modification.

The problem-based learning (PBL) approach can be considered to be an effort towards making learning more dynamic. There are obviously pros and cons to this, with advocates and critics of this approach. Here, we do not wish to discuss the complete reformulation of the curriculum, or to go into the issue of what curricular proposal would be most appropriate, if only because we take the view that different realities should have different teaching proposals. Rather, we would like to discuss how to better integrate basic sciences and to highlight their importance.

In this regard, several authors have drawn attention to the need for better integration of teaching. In 2000, Harden published an interesting article that proposed 11 consecutive and progressively correlated steps that he named the “integration ladder”, which can be used for assessing and planning the medical curriculum.^1^

A very interesting solution was produced by the University of California. In rethinking its medical teaching, it considered that full integration of basic, clinical and social sciences was important. The idea was that the final application of knowledge, which is dependent both on practice and on deepening of the theory, could be better explored by schools through introducing multimodal teaching tools. This thinking resulted in implementation of a program named “Human Biology and Disease”, which basically aimed to unify basic sciences and clinical sciences. This methodology was studied and described in a paper published in 2009.^2^

Another interesting experience at Harvard Medical School was described in 2007. This consisted of a new proposal that sought better integration of the curriculum through not dividing the material into blocks over the academic year, thus ensuring that students were in contact with their different subjects continuously and unceasingly throughout the year.^3^

There is a worldwide trend towards this curricular integration: both horizontally, between subjects, and vertically, between basic sciences and clinical sciences. It is taking place through the argument that it provides teaching that is more complete and effective in terms of knowledge and applicability.

In this regard, article three of the national curricular directives for undergraduate medical courses that have been issued by the Higher Education Chamber of the Brazilian National Education Council states the following:^4^

“*The undergraduate medical course provides entry/professional training for physicians with a profile of generalist, humanist, critical and reflective training, who have the capacity to act, based on ethical principles, on the health-illness process at its different levels of care, with actions to promote health, prevent disease, recover health and rehabilitate the individual, from a perspective of comprehensiveness of care, with a sense of social responsibility and commitment towards active citizenship, as a promoter of full health for human beings*.”

To fulfill this target, medical schools and educators should make every effort to constantly improve teaching. This should, without any doubt, include adaptation of the basic sciences program, thereby making it dynamic, efficient and (why not?) more attractive to students. All of this has the aim that basic sciences should serve as a firm foundation for clinical knowledge and for development of research.
